# Integration of multi-omics data to unveil the molecular landscape and role of piRNAs in early-onset colorectal cancer

**DOI:** 10.1186/s12916-025-04074-2

**Published:** 2025-04-29

**Authors:** Siyun Zhou, Lili Yu, Jianhui Zhao, Qian Xiao, Jing Sun, Lijuan Wang, Yuan Zhou, Yadong Lu, Malcolm G Dunlop, Evropi Theodoratou, Honghe Zhang, Kefeng Ding, Xue Li

**Affiliations:** 1 Department of Colorectal Surgery and Oncology, The Second Affiliated Hospital, School of Public Health, Zhejiang University School of Medicine, Hangzhou, Zhejiang China; 2 Centre for Global Health, Usher Institute, University of Edinburgh, Edinburgh, UK; 3 Department of Colorectal Surgery and Oncology, Key Laboratory of Cancer Prevention and Intervention, Ministry of Education, The Second Affiliated Hospital, Zhejiang University, Hangzhou, Zhejiang China; 4 Department of Pathology, Zhejiang University School of Medicine, Hangzhou, Zhejiang China; 5 Cancer Research UK Scotland Centre and Medical Research Council Human Genetics Unit, University of Edinburgh, Edinburgh, UK; 6 Center for Medical Research and Innovation in Digestive System Tumors, Ministry of Education, Hangzhou, China; 7 Zhejiang Provincial Clinical Research Center for CANCER, Hangzhou, China

**Keywords:** Early-onset colorectal cancer, Multi-omics, PIWIL1, PiRNA, Biomarker

## Abstract

**Background:**

The incidence of early-onset colorectal cancer (EOCRC) (< 50 years) has been steadily rising, with a parallel increase in metastatic and invasive cases. To elucidate the molecular mechanisms underlying this aggressive phenotype, we performed comprehensive multi-omics profiling to delineate the distinct features of EOCRC, with a focus on key drivers of metastatic and invasive potential.

**Methods:**

We initially characterized the genome, epigenome, and transcriptome of tumors from 515 (69 EOCRC and 446 late-onset CRC [LOCRC]) cases in The Cancer Genome Atlas. Key candidate molecules were further validated using RNA-seq and scRNA-seq data. Multi-omics profiling revealed PIWIL1/piRNA as a hallmark of EOCRC, with further validation through in vitro functional assays, transcriptomic profiling, and Kaplan-Meier survival analysis.

**Results:**

EOCRC demonstrated a mutational landscape similar to that of LOCRC, with comparable oncogenic driver mutations and somatic copy-number alterations. However, EOCRC exhibited a higher frequency of deletion in chromosomes 6, 15, and 19 regions, along with metabolic reprogramming favoring aerobic glycolysis and lipid metabolism. Integrative transcriptomic and DNA methylation analyses identified six EOCRC-specific molecules, including PIWIL1. Notably, PIWIL1 was mainly expressed in epithelial cells, with lower expression in EOCRC versus LOCRC. Its downstream piRNAs (FR019019, FR019089, and FR132045) were also downregulated in EOCRC. Functional experiments demonstrated that FR019089/FR019019 overexpression suppressed migration and invasion. Clinically, low FR019089 levels correlated with significantly shorter progression-free and overall survival in EOCRC patients. Additionally, downstream pathways of FR019089 and FR019019 overexpression were enriched in anti-cancer-related signaling pathways.

**Conclusions:**

Our multi-omics approach yields novel insights into the molecular underpinnings of EOCRC and we characterize the role of PIWIL1-associated piRNAs in modulating EOCRC metastasis and invasion. FR019089 shows promise as a prognostic biomarker with potential clinical utility in the risk stratification and management of EOCRC patients.

**Graphical Abstract:**

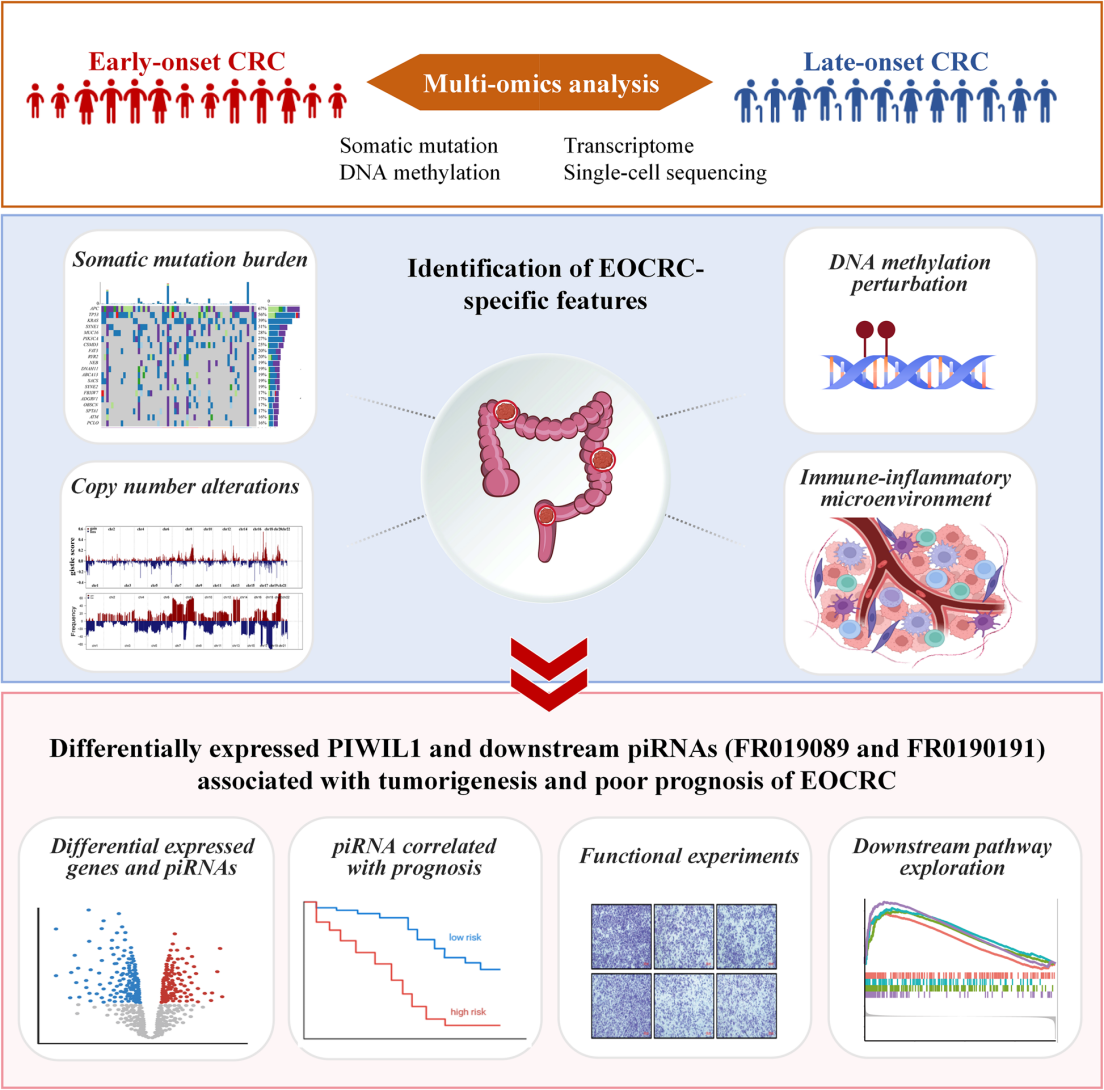

**Supplementary Information:**

The online version contains supplementary material available at 10.1186/s12916-025-04074-2.

## Background

Colorectal cancer (CRC) ranks as the third most diagnosed cancer and the second leading cause of cancer-related mortality worldwide, representing a significant global health burden [1]. Despite a global decrease in CRC incidence over the last 40 years, early-onset colorectal cancer (EOCRC, defined by a diagnosed age under 50 years) has been steadily increasing at an alarming rate of 2–4% annually [2–5]. The EOCRC tends to be localized in the distal colon and rectum [6]. Owing to the lack of routine screening in younger patients and the inherently more aggressive features of EOCRC tumors, a larger proportion (85%) of EOCRC patients are symptomatically at diagnosis, with a significantly higher prevalence of advanced-stage disease than the overall CRC population (50%) [6, 7].

Environmental factors have been implicated in EOCRC pathogenesis, yet the molecular changes driving its widespread rise remain elusive [2, 8, 9]. Notably, EOCRC exhibits distinct molecular alterations compared to late-onset CRC (LOCRC). A few studies have attempted to profile the somatic mutational landscape of EOCRC patients, revealing that somatic mutations in oncogenic driver genes differ quite a bit between EOCRC and LOCRC [7, 10]. A significant mutation frequency in PIK3R1, PDGFRA, FLT3, and KDR gene mutations has been identified [6]. Another study integrated deconvolution or mutational signatures of EOCRC and epidemiologic data to uncover mutagenic processes that contribute to tumorigenesis across the age continuum [11]. However, existing multi-omics studies on EOCRC remain largely descriptive, with limited functional validation of key molecular drivers implicated by omics data.

Beyond genomics and epigenomics, transcriptomic studies have identified mRNA-based signatures for EOCRC [12–14]. Nevertheless, the precise involvement of key non-coding RNAs (ncRNAs), such as PIWI-interacting RNAs (piRNAs), in EOCRC remains poorly understood. Recently, evidence gathered has found that small ncRNAs (including piRNAs) are closely related to the occurrence and progression of cancers, highlighting their potential as diagnostic or prognostic biomarkers [15]. Piwi Like RNA-Mediated Gene Silencing 1 (PIWIL1), associated with a class of small ncRNAs called piRNAs of 20–35 nt, plays a crucial role in somatic cell function and increased CRC progression [16]. PIWIL1 overexpression correlates with poor tumor differentiation, infiltration, lymph node invasion, metastasis, and reduced overall and disease-free survival in CRC [17]. Nevertheless, detailed exploration of PIWILs and piRNAs in CRC, especially in EOCRC, remains limited, leaving the cellular pathways and molecular mechanisms involving somatic piRNAs poorly understood.

The study identified distinct genomic and epigenomic alterations in EOCRC through integrated multi-omics analysis, demonstrating the power of multi-omics approaches in elucidating age-specific tumor heterogeneity. Building on these findings, we investigated the functional mechanisms by which key piRNAs mediate the enhanced migratory and invasive properties in EOCRC.

## Methods

### Study design

This study integrated multi-dimensional omics data, including whole-genome sequencing, bulk/single-cell RNA-seq, and DNA methylation profiling. Genomic data were utilized to characterize the mutational status and somatic copy number variations (CNVs) related to EOCRC and LOCRC. Based on transcriptome data, we elucidated the feature of tumor microenvironment and metabolic shifts related to age of onset CRC. To probe into the EOCRC-specific molecular events, we analyzed the differentially expressed genes (DEGs) at transcriptional level and differentially methylated CpGs (DMCs) at the epigenetic level; pivotal genes were then validated through integrating the DNA methylation and gene expression data. Single-cell RNA-sequencing data were further analyzed to specify the cell types with expression of the key candidate genes in CRC tumors. Based on multi-omics findings, we investigated the functional role of PIWIL1/piRNA through in vitro experiments.

### Patients and clinical tissue samples

Multiple omics datasets were obtained from two databases, namely the TCGA [18] (*N* = 515) and the Gene Expression Omnibus (GEO) datasets under accession code GSE39582 [19, 20] (*N* = 585), GSE17536 [21, 22] (*N* = 177), and GSE17537 [21, 23] (*N* = 55). The genomics, transcriptomics, epigenomics, and clinical information of 515 European individuals with CRC (a combination of COAD and READ) from the TCGA were downloaded. To validate the expression of candidate biomarkers for EOCRC, expression profiles from three GEO datasets were extracted. We downloaded scRNA-seq data (GSE132465) [24, 25], comprising 23 primary tumor samples from EOCRC patients and 10 matched normal mucosa samples. The datasets mentioned above were downloaded from several websites (https://portal.gdc.cancer.gov/; http://www.cbioportal.org/; https://www.ncbi.nlm.nih.gov/geo/).

The acquisition of all tissue samples utilized in this research was conducted following the approval granted by the Biomedical Ethics Committee of Zhejiang University (ZGL202306-3), with each patient providing written informed consent. A total of 18 EOCRC patients who were firstly diagnosed with CRC were recruited from the First Affiliated Hospital of Zhejiang University School of Medicine between 1 st Mar, 2023 and 19 th Feb, 2024. Eighteen pairs of colorectum tumor and control biopsy specimens were obtained during laparoscopic surgery. The control tissue was collected at least 2 cm from the edge of the CRC tissue. Moreover, all CRC and control tissues were examined and evaluated by at least three pathologists. The research was conducted following recognized ethical guidelines.

### Genomic data processing

The mutation annotation format file with aggregated mutation of CRC cases was obtained from the TCGA portal. Candidate somatic single-nucleotide variants were identified using Mutect algorithms. To identify significantly mutated genes, we used the “maftools” R package to analyze the MAF files of the TCGA mutation data. The “lollipopPlot” function was applied to display mutation types in the two different subgroups, and results were visualized using Lollipop charts. GISTIC software was widely applied to detect both broad and focal (potentially overlapping) recurring events. We used GISTIC 2.0 [26] to identify genes exhibiting significant amplification or deletion. The parameter threshold was defined as amplification or deletion length > 0.1 and *P* < 0.05.

### Identification of differential genes and differentially methylated CpG sites

We defined protein-coding genes differentially expressed between EOCRC and LOCRC tissue samples using the “edgeR” package on log_2_-transformed gene expression levels (quantified by transcript per million). Additionally, to account for differences in the distribution of baseline characteristics between EOCRC and LOCRC, the inverse probability of treatment weighting (IPTW) using propensity scores was performed to balance baseline covariates associated with each group [27]. Gender and stage at diagnosis were included in the IPTW process as covariates. By this approach, all patients in each group contributed to the final analysis with a specific calculated weight. Weighted gene expression data was analyzed to support the stability of DEGs results without balancing baseline covariates. Genes with an absolute log_2_-fold change (FC) ≥ 1 and false discovery rate (FDR) < 0.05 in comparison were considered DEGs. GO analysis was conducted using the enrichGO function implemented in the R package “clusterProfiler” (v. 3.8.1) [28].

DNA methylation array data (Illumina Infinium HumanMethylation450 BeadChip, HM450) were obtained from the GDC Data Portal, which interrogated 485,764 cytosine positions of the human genome, out of which 482,421 positions (99.3%) were CpG dinucleotides [29]. We applied the following criteria for quality control: (i) probes with mean detection *P* value ≥ 0.01 in > 5% of samples were removed from all samples; (ii) probes on the X or Y chromosome were removed; (iii) probes overlapping with single-nucleotide polymorphisms were removed; (iv) probes mapped to multiple sites in the human genome were removed. Finally, 308,242 probes were kept for further analysis. The CpG probe annotation file was downloaded from the ENCODE Project database (http://genome.ucsc.edu/ENCODE/downloads.html). Each CpG probe was annotated with the corresponding gene [30]. The package “limma” (implemented in “ChAMP” packages [31]) was used to identify DMC sites between EOCRC and LOCRC patients. A linear model was conducted to calculate the *P* value for differential methylation, and CpG probes meeting the criteria of *β* value ≥ 0.1 and FDR < 0.05 were defined as significant differential CpGs.

### Correlation analysis between DNA methylation and gene expression

The correlations between the methylation levels of DMCs and the expression levels of their corresponding genes were examined using Spearman’s rank correlation. To obtain the DNA methylation driver genes, we considered FDR < 0.05 and the absolute Spearman rank correlation coefficient |*r*|> 0.30 as a statistically significant correlation. We further considered the consistency of direction between the methylation level of DMCs and the corresponding gene expression level for a more rigorous screening. DMCs located in promoters were selected in this work, and we applied the following criteria: (i) Wilcoxon rank-sum test was used to detect DNA methylation differential genes with *P* < 0.05. (ii) Negative correlations (Spearman *r* < − 0.30, FDR < 0.05) between DMCs and their corresponding genes.

### piRNA transcriptome data collection and processing

To identify the expression levels of candidate piRNAs between EOCRC and LOCRC tissues, we obtained the piRNA expression matrix of 341 CRC patients (44 EOCRC patients and 297 LOCRC patients) from the TCGA dataset, which was characterized by Martinez et al. [32]. piRNAs with an absolute log_2_FC ≥ 1 and FDR < 0.05 were considered EOCRC-specific piRNAs using the “edgeR” package.

### Pathway enrichment and immune signature analysis

GSEA was performed using the “GSVA” package [33]. Enrichment results with a criterion of FDR < 0.05 were considered significant. Gene sets were derived from the Broad Institute’s Molecular Signatures Database (MsigDB). In addition, the fatty acid-associated genes and glycolysis-associated genes were obtained from the hallmark, reactome, and kegg gene sets listed in MsigDB. Kyoto Encyclopedia of Genes and Genomes (KEGG) enrichment analyses were performed using the R package “clusterProfiler.” The CIBERSORT platform (https://cibersort.stanford.edu/) was used to estimate immune cell proportions based on the LM22 reference profile. Computing gene set scores for representative immune signatures (immune checkpoint, immunosuppression modulators for TC-6, immune response to tumor cells, etc.) was performed using the “ssGSEA” algorithm.

### Single-cell transcriptome analysis

We followed the pipeline to process the dataset [24]. Briefly, the sequencing data was aligned with the human reference genome (GRCh38) and processed using the CellRanger 2.1.0 pipeline (10 × Genomics). The original gene expression matrix was filtered using the “Seurat” package [34] based on the cell counts with > 1000 unique molecular identifier (UMI) counts, > 200 genes and < 6000 genes, and > 20% mitochondrial gene expression in UMI, which filtered gene expression matrix with 67,296 cells. After defining the global cell type, cells with several genes exceeding the outliers were removed to eliminate potential doublets, which generated a final gene expression matrix with 63,689 cells available in this study. The GSE132465 dataset contained annotated cell types from each sample. To eliminate cells of ambiguous identity, we determined diverse cell types in the GSE132465 dataset, and the “SingleR” package was used for initial clustering and cell type identification. T cells, B cells, epithelial cells, stromal cells, myeloid cells, and endothelial cells were especially recognized by *t*-distributed stochastic neighbor embedding (t-SNE) in the “Seurat” R package. The raw counts were then normalized and transformed to the natural log scale for subsequent analysis.

### Cell culture and treatment

Human CRC cell lines (SW620, SW480, DLD-1, HT29, HCT116, HCT8, and RKO) and human normal intestinal epithelial cell line (NCM460) were purchased from the American Type Culture Collection (Manassas, VA, USA). SW620, SW480, DLD-1, HT29, HCT116, HCT8, and NCM460 were grown in complete RPMI- 1640 medium (VivaCell Biosciences, Shanghai, China). RKO was cultured in complete DMEM medium (VivaCell Biosciences). All media were supplemented with 10% FBS (Gibco, Gaithersburg, MD) and penicillin/streptomycin (NCM Biotech, Suzhou, China). Cells were grown in a humidified atmosphere at 37℃ with 5% CO_2_. FR019089 mimic, FR019089 inhibitor, FR019019 mimic, and FR019019 inhibitor were purchased from GenePharm (Shanghai, China). Transfection of mimic and inhibitor was conducted using GenMute siRNA Transfection Reagent (SignaGen Laboratories, Jinan, China).

### RNA isolation, qRT-PCR, RNA-seq, and analysis

Total RNA from cells and tissues was isolated using the Trizol reagent (Invitrogen, Carlsbad, CA, USA). For qRT-PCR, total RNA (500 ng) was used to synthesize cDNA via the miRNA 1 st Strand cDNA Synthesis Kit (Vazyme Biotech, Nanjing, China, #MR101), and the miRNA Universal SYBR qPCR Master Mix (Vazyme Biotech, #MQ101) was used to conduct the amplification reactions according to protocols. The primers for qRT-PCR are listed in Additional file 1: Table S1.

For RNA-seq, total RNA was sequenced on an Illumina Novaseq™ 6000 (LC-Bio Technology CO., Ltd., Hangzhou, China) following the vendor’s recommended protocol. Raw Illumina sequencer output was converted to FASTQ format and was aligned to the Homo sapiens GRCh38 using the HISAT2 (https://ccb.jhu.edu/software/hisat2). The StringTie was used to estimate the expression levels of all transcripts by calculating FPKM. The EdgeR package was applied to analyze DEGs (absolute log_2_FC ≥ 1 and FDR < 0.05). GSEA was performed using “GSVA” packages [33]. Enrichment results with a criterion of qvalue < 0.05 were considered significant. Volcano plots were created using the “pheatmap” package. RNA sequencing data is available upon request.

### CCK8 assay and colony formation assay

For the CCK8 assay, 2000 cells were plated in a 96-well microplate. After 0, 24, 48, and 72 h of proliferation, 10 μL of CCK8 (Biosharp, Hefei, China) was added to each well for 1 h, and cell viability was detected at 450 nm. For the short-term colony formation assay, 5000 cells were seeded in 12-well plates. After 1 week of proliferation, cells were fixed in 4% methanol and stained with crystal violet to count cell number. Three independent experiments were performed, and ImageJ software was used for quantification.

### Transwell migration and invasion assay

Transwell and matrigel chamber plates (Corning Costar, New York, USA) were utilized to measure cell motility and invasiveness. 150,000 cells for RKO and 70,000 cells for HCT8 were loaded into a transwell cultured with serum-free media on the upside of the membrane, and a complete medium with 10% FBS was added at the bottom of the insert. Cells were led to adhere and transmigrate through the membrane at 37 °C, 5% CO_2_. After 28 h for RKO and 17 h for HCT8, transmigrated cells on the lower surface of the membrane were fixed in 4% methanol and stained with crystal violet. Images of cells on the lower face of the filter were captured in fields at × 10 magnification. Independent experiments were performed in triplicate. ImageJ software was used for quantification.

### Statistical analysis

The Wilcoxon rank-sum test, *t*-test, and Kruskal-Wallis test were used for quantitative data comparisons, and Fisher’s exact test and $\chi^2$ test were used to assess the difference in qualitative data. To investigate the prognostic potential of candidate piRNAs, Kaplan–Meier analysis and log-rank test were used to estimate and compare OS and PFS rates among different subgroups stratified by piRNA expression (high versus low level). A log-rank test *P* < 0.05 was used to define differences in survival time. For wet experiments, statistical analyses were carried out using GraphPad Prism software (version 8.0). For two- or three-group comparisons, Student’s *t* test or one-way ANOVA followed by Dunnett’s test was used and the figure legends specified the statistical tests used for each data. Graphs represent mean ± standard deviation (SD) unless otherwise stated. All *P*-values were two-sided, and *P* < 0.05 was considered statistically significant.

## Results

### Overview of clinical features and genomic alterations of EOCRC

**Fig. 1:**
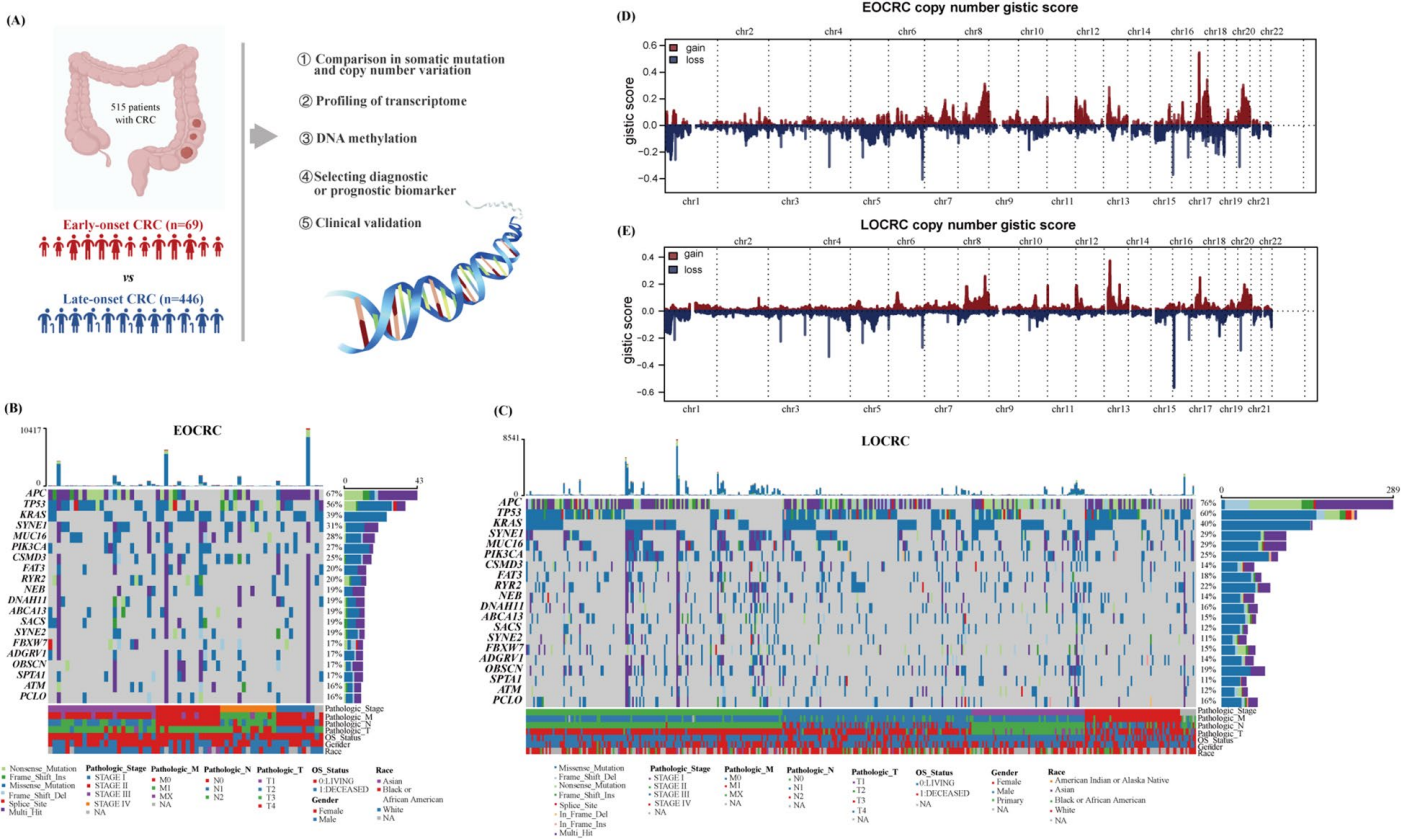
Population features and genomic alterations of EOCRC and LOCRC.** A** A total of 69 EOCRC and 446 LOCRC patients are enrolled in the TCGA cohort. The study diagram in the TCGA cohort is comprised of a comparison in somatic mutation and CNVs, profiling of transcriptome and DNA methylation, selection of diagnostic or prognostic biomarkers, and clinical variation. **B,C** Somatic mutations of the top mutated genes among different subtypes in EOCRC (**B**) and LOCRC (**C**). Genes are ordered by the total mutation frequencies. **D,E** The distribution of CNVs features across chromosomes for EOCRC (**D**) and LOCRC (**E**). TCGA, The Cancer Genome Atlas; EOCRC, early-onset colorectal cancer; LOCRC, late-onset CRC; CNV, copy number variation

In the TCGA cohort, a group of CRC patients provided tumor tissues for multi-omics profiling (Fig. 1A), and a total of 69 (13.40%) EOCRC and 446 (86.60%) LOCRC patients with summarized demographic characteristics in Additional file 1: Table S2 were included in this study. Patients with EOCRC were more frequently initially diagnosed with stage III (40.58% vs. 27.80%) or stage IV (20.29% vs. 13.68%). The prevalence of low microsatellite instability (MSI-low) tumors was higher among patients with EOCRC (23.19% vs. 15.93%), whereas the difference in MSI-high status between EOCRC and LOCRC was not observed (13.04% vs. 13.90%). Next, mutational analysis revealed that APC, TP53, and KRAS were the most frequently mutated genes (Fig. 1B, C), consistent with prior studies [7, 10]. Although APC mutations were less common in EOCRC than in LOCRC (67% vs. 76%), their mutation types and distribution differed markedly between subgroups (Additional file 2: Figure S1). CNV analysis showed amplifications in chromosomes 8, 13, and 17 in both groups, while deletions in chromosomes 6, 15, and 19 were more prevalent in EOCRC (Fig. 1D, E). Collectively, these results highlight distinct genomic alterations and molecular characteristics in EOCRC.

### Shift of immune microenvironment and cellular metabolism in EOCRC

**Fig. 2:**
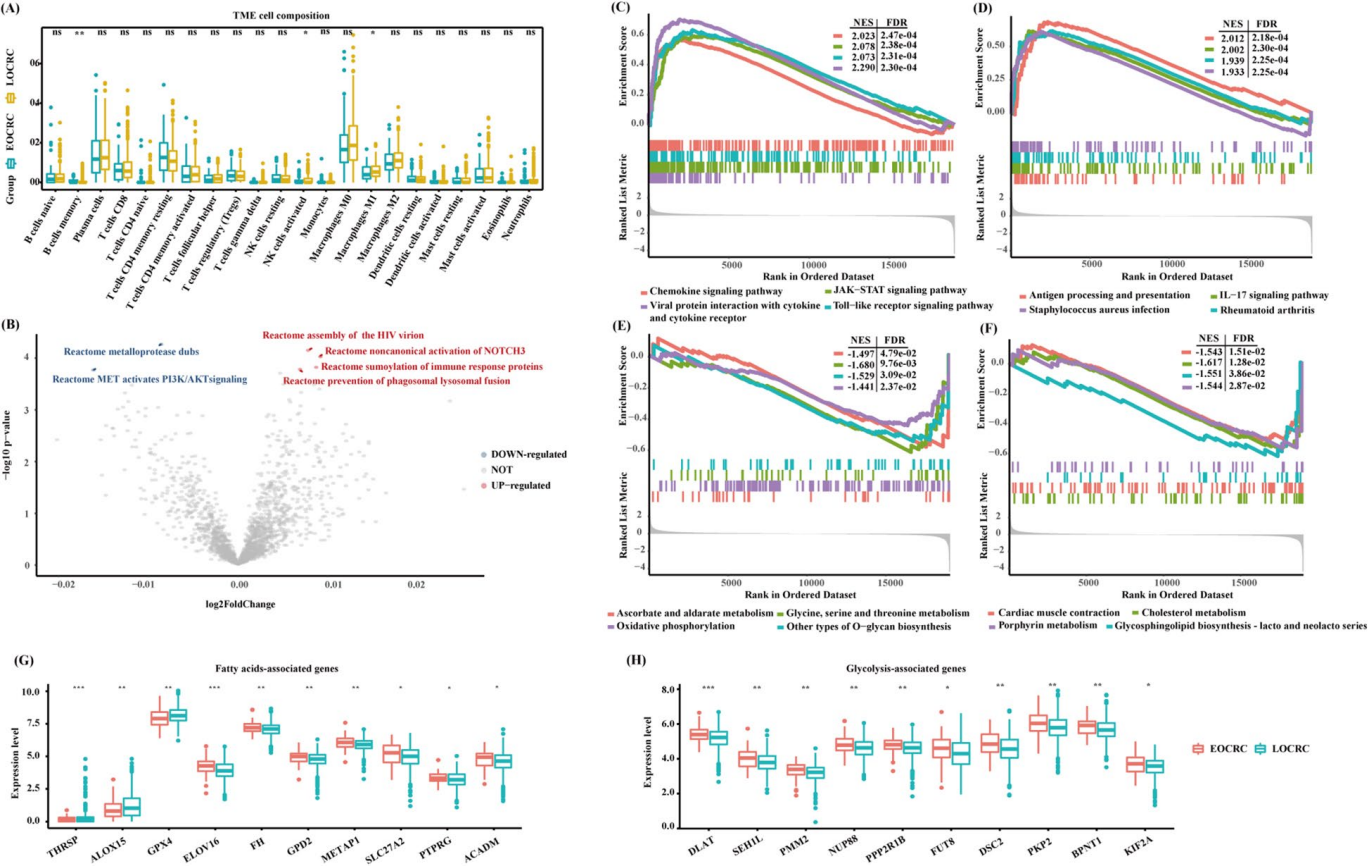
Enrichment pathways of EOCRC.** A** The cell proportions in tumor microenvironment of EOCRC and LOCRC groups. *P < 0.05; **P < 0.01; ***P < 0.001; ****P < 0.0001, significant statistical differences between the two subgroups were assessed using the Wilcoxon test. **B** Volcano plot indicates the comparison at transcriptomic levels between EOCRC and LOCRC through ssGSEA enrichment scores. Upregulated pathways in the EOCRC group are shown in red and downregulated pathways are shown in blue. **C–F** GSEA results for the upregulated (**C,D**) and downregulated (**E,F**) pathways in the EOCRC group and LOCRC group, respectively (FDR < 0.05). **G,H** Quantification of selected genes of the fatty acids pathway (**G**) and glycolytic pathway (**H**) in the TCGA cohort (log_2_ intensity levels). Each boxplot shows the median and interquartile range (25 th–75 th percentiles). *P < 0.05, **P < 0.01, ***P < 0.001, significance was determined using a Wilcoxon rank-sum test. EOCRC, early-onset colorectal cancer; LOCRC, late-onset CRC; ns, not significant; GSEA, gene-set enrichment analysis; NES, normalized enrichment score

The immune system is widely recognized as a critical factor in CRC onset and progression [35], and the tumor immune microenvironment has emerged as an independent biomarker of immunotherapy efficiency. To elucidate the feature of the tumor microenvironment of EOCRC, we utilized the single sample gene set enrichment analysis (ssGSEA) to quantify the abundance of 22 immune cell types. The tumor region of EOCRC was significantly enriched with memory B cells, whereas decreasingly enriched in NK cells and M1 macrophages (Fig. 2A). The significant pathways included pathways related to cancer-associated pathways and immune response (Fig. 2B). Interestingly, further GSEA analysis revealed upregulated cancer-associated pathways, including chemokine signaling pathway, JAK-STAT signaling pathway, IL-17 signaling pathway, and bacterial-related pathways (FDR < 0.05; Fig. 2C, D and Additional file 1: Table S3). In contrast, the downregulated pathways were mainly enriched in metabolic pathways, including ascorbate and aldarate metabolism, glycine, serine, and threonine metabolism, oxidative phosphorylation, cholesterol metabolism, and glycosphingolipid biosynthesis pathways (FDR < 0.05; Fig. 2E, F and Additional file 1: Table S3). Altogether, these results indicate the existence of an immune-inflammatory microenvironment in the tumor region, which is consistent with previous studies [36]. To assess metabolic differences, we analyzed fatty acid oxidation and glycolysis, finding significantly elevated expression of associated genes in EOCRC (Fig. 2G, H), indicating a shift toward aerobic glycolysis and lipid metabolism that may drive aggressive tumor biology.

### Differentially expressed PIWIL1 associated with tumorigenesis of EOCRC

**Fig. 3:**
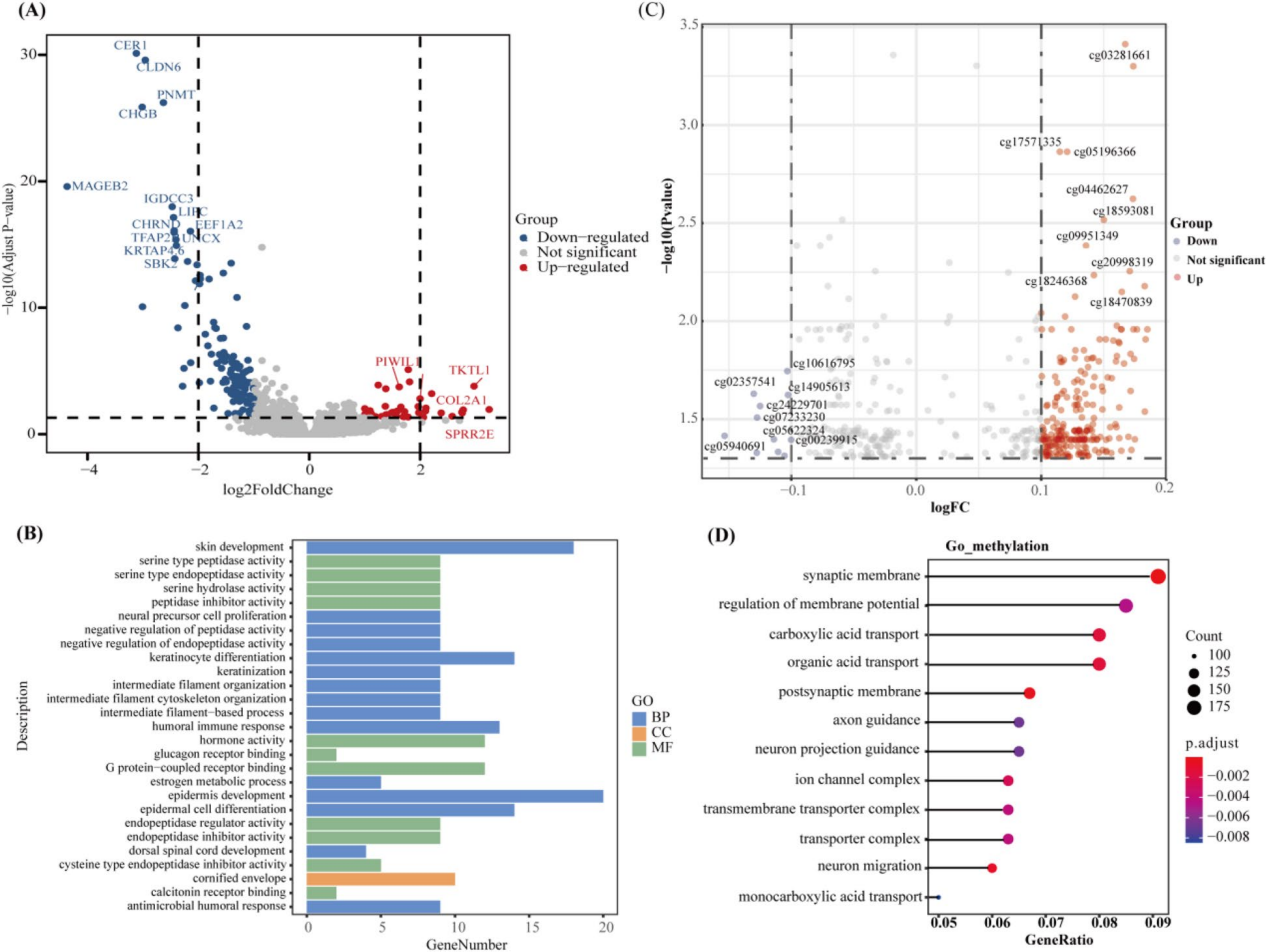
Gene expression and epigenetics difference of EOCRC and LOCRC tumors.** A** Volcano plot illustrates the differentially expressed genes in LOCRC. Upregulated genes in LOCRC and EOCRC patients are shown in red and blue, respectively. **B** Gene Ontology enrichment analysis for the differential expression genes. **C** Volcano plot indicates differentially methylated CpGs between EOCRC and LOCRC. Up-methylated CpGs in LOCRC and EOCRC patients are shown in red and blue, respectively. **D** Gene Ontology enrichment analysis for the CpG-mapped genes. EOCRC, early-onset colorectal cancer; LOCRC, late-onset CRC

We then probed into the EOCRC-specific molecular events at both transcriptional and epigenetic levels. Transcriptomic analysis identified 168 DEGs in LOCRC versus EOCRC (40 upregulated and 128 downregulated; Fig. 3A). Pathway enrichment revealed the top 27 significant gene ontology (GO) terms (*P* < 0.05, Fig. 3B), including cell function and the nervous system pathways. More DEGs were analyzed using weighted gene expression data (81 upregulated and 180 downregulated; Additional file 1: Table S4), which contained all the molecules analyzed above. At the epigenetic level, DNA methylation profiling detected 241 significant DMCs (230 hypermethylated and 11 hypomethylated) in LOCRC compared to EOCRC (Fig. 3C). Genes harboring these DMCs were enriched in 12 GO pathways (*P* < 0.05, Fig. 3D), involving carboxylic acid, monocarboxylic acid, and organic acid transport.

The role of DNA methylation in gene regulation is multifaceted, but methylation in promoters is often considered the hallmark of gene repression [37]. Through integrated analysis of transcriptome and DNA methylation data, we consistently identified six key modules: PIWIL1, pyroglutamylated RFamide peptide receptor (QRFPR), carbohydrate sulfotransferase 8 (CHST8), GATA binding protein 4 (GATA4), membrane-spanning 4-domains A1 (MS4A1), and Wnt family member 3 A (WNT3A). Significant negative correlations were observed between promoter DMCs and gene expression levels (PIWIL1, *r* = − 0.54, *P* = 4.14e − 27; CHST8, *r* = − 0.20, *P* = 1.76e − 04; QRFPR, *r* = − 0.39, *P* = 5.83e − 14; GATA4, *r* = − 0.44, *P* = 1.07e − 17; MS4A1, *r* = − 0.13, *P* = 0.014; WNT3A, *r* = − 0.10, *P* = 0.065; Additional file 2: Figure S2 and Additional file 1: Table S5). These results highlight that in a variety of molecular processes, the promoter DNA hypermethylation, and hypomethylation regulate gene expression, which may be underneath the initiation and progression of EOCRC.

**Fig. 4:**
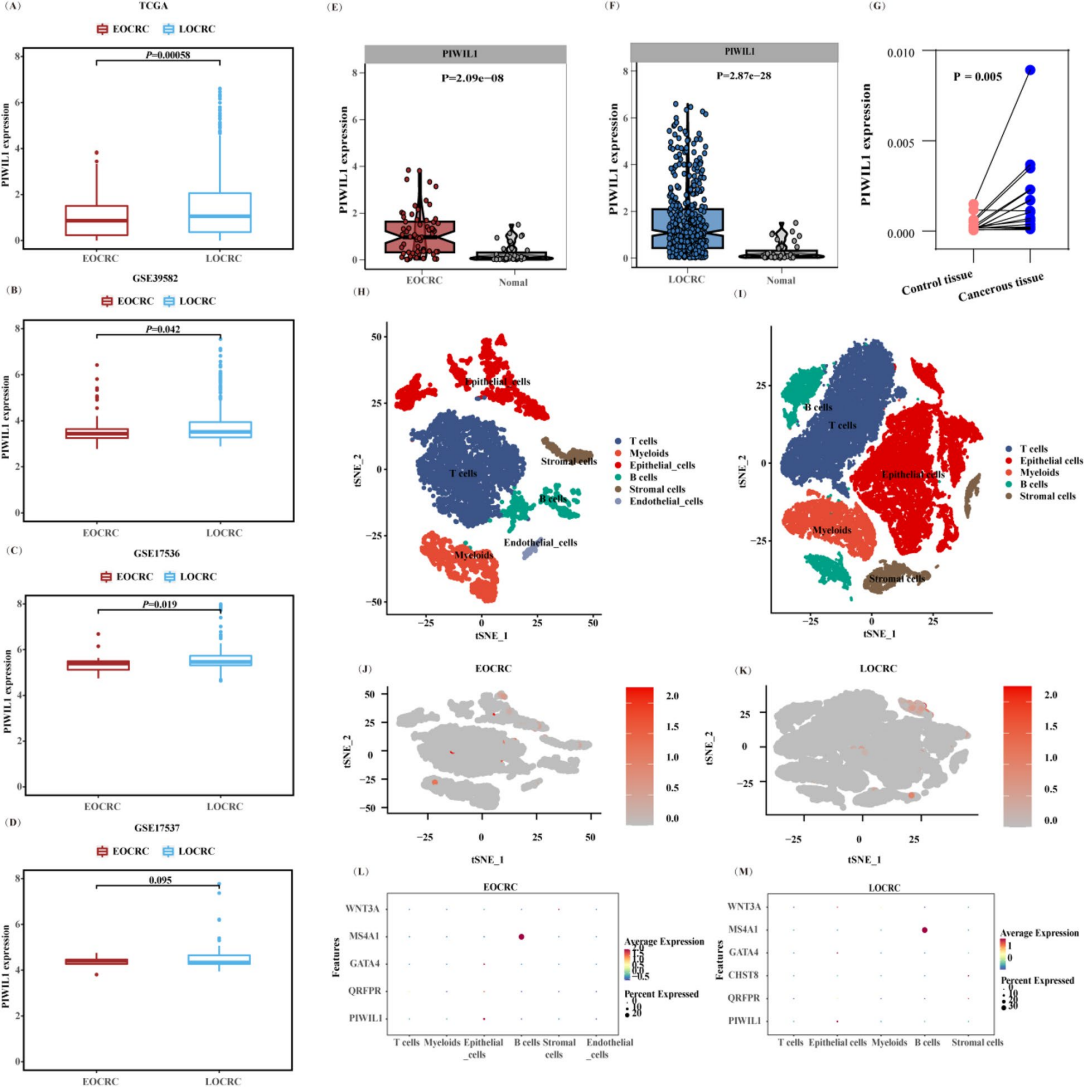
Validation of differentially expressed PIWIL1 in different datasets.** A–D** The expression of PIWIL1 in EOCRC and LOCRC patients from the TCGA (**A**), GSE39582 (**B**), GSE17536 (**C**), and GSE17537 (**D**) datasets. **E–G** Differential expression analysis of PIWIL1 between normal and EOCRC (**E**) or LOCRC (**F**) tissues from the TCGA cohort and clinical specimens (**G**). Significance was determined using a Wilcoxon rank-sum test. **H,I** UMAP plots of unsupervised clustering result of tumor subclusters in EOCRC (**H**) and LOCRC (**I**) from the GSE132465 dataset. The tSNE plots show single cells of CRC tissues across six major cell types. **J,K** The expression pattern of PIWIL1 in single-cell level in EOCRC (**J**) and LOCRC (**K**) tissues. Cells expressing PIWIL1 are colored red in the tSNE plots using the normalized value. **L–M** Bubble plots of the average and percent expression of PIWIL1 in different cell subtypes of EOCRC (**L**) and LOCRC (**M**) tissues. EOCRC, early-onset colorectal cancer; LOCRC, late-onset CRC; tSNE, t-distributed stochastic neighbor embedding

PIWIL1 belongs to the Piwi clade of the Argonaute family, mainly expressed in germ cells but absent in somatic tissues [38]. Overexpression of PIWIL1 is common to several tumor types (e.g., hepatocellular carcinoma (HCC) [39]), and its aberrant expression has been associated with tumorigenesis and poor prognosis [40]. Therefore, we first validated its expression pattern in CRC using TCGA and three external datasets (GSE39582, GSE17536, and GSE17537). As shown in Fig. 4A–D, the transcription level of PIWIL1 was significantly lower in EOCRC compared with LOCRC (*P* < 0.05). Further comparison of PIWIL1 expression in EOCRC, LOCRC, and healthy tissues using the TCGA data and clinical specimens (a total of 18 pairs of colorectum tumor and control specimens; baseline information of the CRC patients is shown in Additional file 1: Table S6) found that PIWIL1 was upregulated in both EOCRC and LOCRC tissues (Fig. 4E–G). Single-cell analysis of the tumor microenvironment revealed predominant PIWIL1 expression in epithelial cells, with significantly higher levels in LOCRC (Fig. 4H–M), particularly in CMS1 and CMS3 subtypes (Additional file 2: Figure S3). These findings suggest PIWIL1 as a key regulator of molecular heterogeneity between EOCRC and LOCRC.

### FR019089 and FR019019 as anti-oncogenic piRNAs were involved in the progression and metastasis of EOCRC

**Fig. 5:**
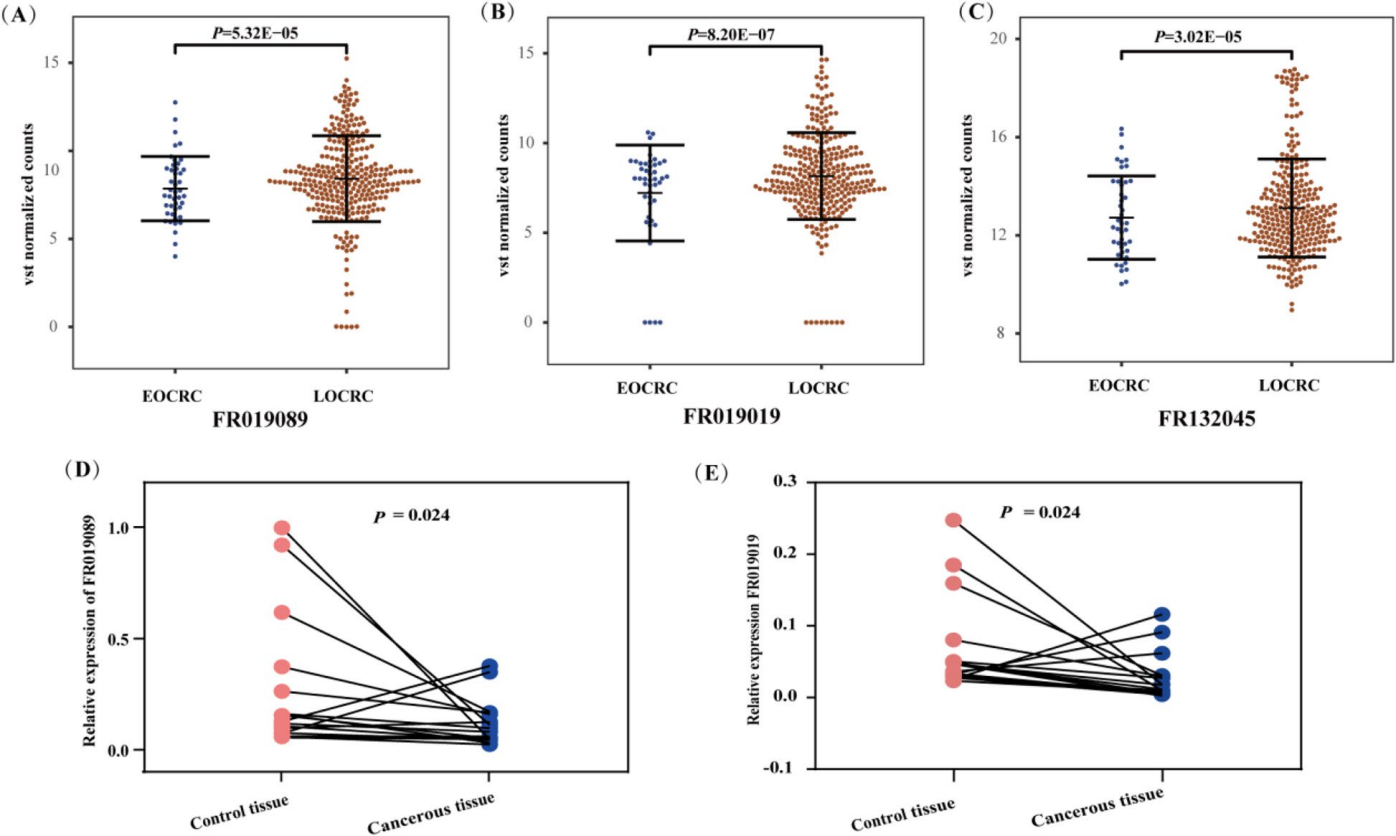
FR019089 and FR019019 are EOCRC-specific piRNAs.** A–C** Differentially expressed FR019089 (**A**), FR019019 (**B**), and FR132045 (**C**) between EOCRC and LOCRC patients from the TCGA datasets. **D,E** qRT-PCR detection of FR019089 (**D**) and FR019019 (**E**) in a subset of 18 cancerous and control tissues from patients of colorectal cancer. U6 was used as an internal control. Statistical analyses were performed by two-tailed Student’s *t* test. EOCRC, early-onset colorectal cancer; LOCRC, late-onset CRC

Since PIWI proteins are central to piRNA biogenesis, we then examined the expression patterns of piRNAs in EOCRC and LOCRC samples. Three differentially expressed piRNAs (FR019019, *P* = 8.20e − 07; FR019089, *P* = 5.32e − 05; FR132045, *P* = 3.02e − 05; Fig. 5A–C) were identified as lower-expressed in EOCRC compared with LOCRC. These piRNAs can be retrieved in piRBase, piRNAdb, and RNAcentral (IDs and RNA sequences in Additional file 1: Table S7). To investigate their uncharacterized roles in CRC, we firstly examined their basal levels in SW620, SW480, DLD-1, HT29, HCT116, HCT8, RKO, and NCM460 cells. FR019089 and FR019019 showed relatively consistent expression patterns in the cell lines mentioned above (Additional file 2: Figure S4 A-C). Furthermore, results of qRT-PCR confirmed the under-expression of FR019089 and FR019019 in CRC tissues compared to the control specimens (Fig. 5D,E), indicating FR019089 and FR019019 as EOCRC-related anti-oncogenic piRNAs that may be involved in CRC progression and metastasis.

### FR019089 and FR019019 inhibit migration and invasion in CRC cells

**Fig. 6:**
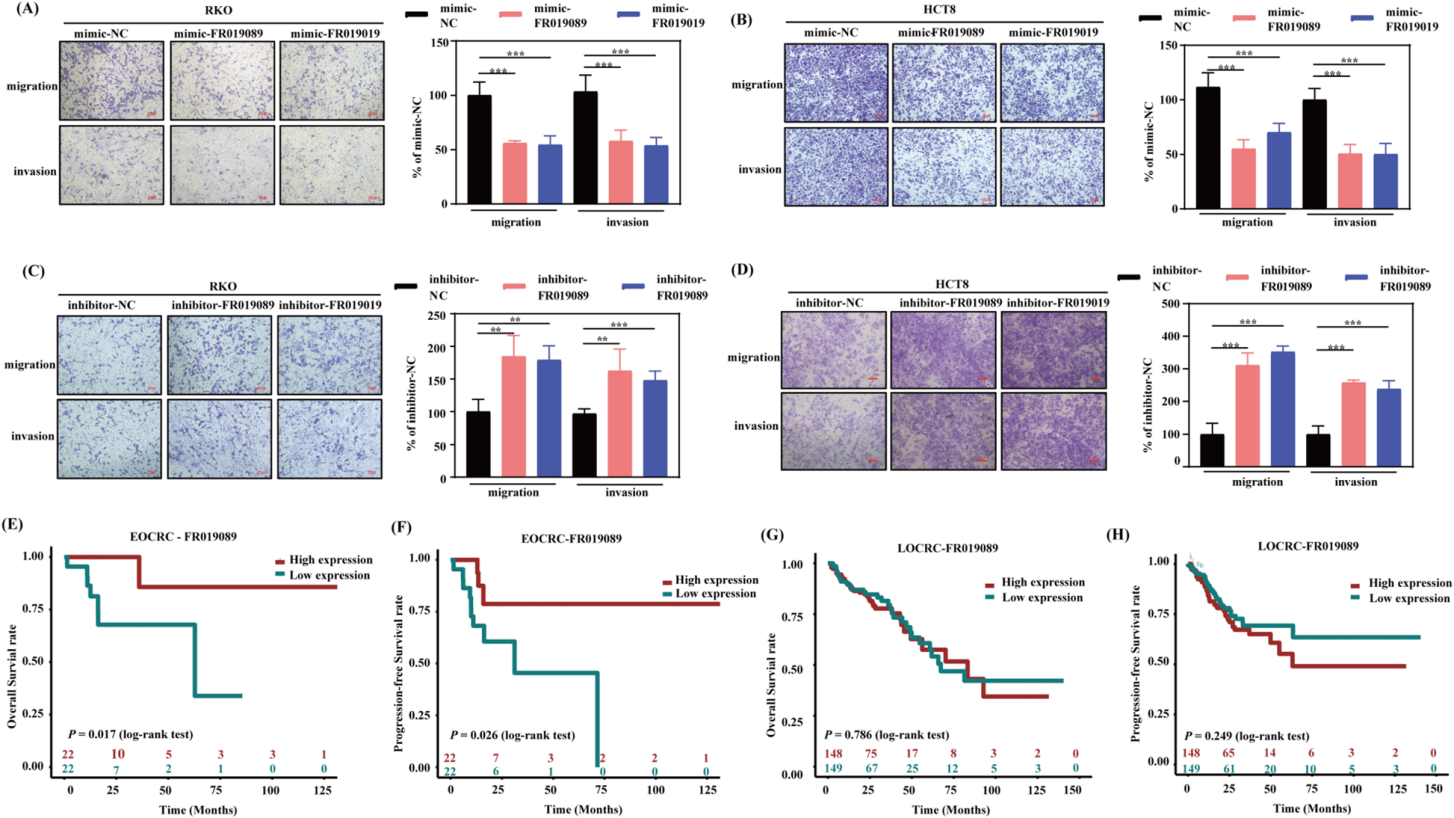
FR019089 and FR019019 attenuate migration and invasion in colorectal cancer cells and the prognostic significance of FR019089 in CRC patients.** A,B** RKO (**A**) and HCT8 (**B**) cells were transfected with either mimic-NC, -FR019089, or -FR019019, transwell assay was tested for the capability of migration and invasion. **C,D** RKO (**C**) and HCT8 (**D**) cells were transfected with either inhibitor-NC, -FR019089, or -FR019019, and transwell assay tested for the capability of migration and invasion. The experiment was repeated thrice independently with similar results. Bar = 100 μm. The quantitative results are presented as the mean ± SD. Statistical analyses were performed by one-way ANOVA with Dunnett’s test. **P < 0.001, ***P < 0.0001 *vs.* the mimic-NC or inhibitor-NC group. **E–H** The overall survival and progression-free survival analysis was performed by the Kaplan–Meier test and log-rank method in EOCRC (**E,F**) and LOCRC (**G,H**) patients, respectively. EOCRC, early-onset colorectal cancer; LOCRC, late-onset CRC

To further investigate the molecular function of FR019089 and FR019019, RKO and HCT8 cells were transfected with the mimics and inhibitors for FR019089 and FR019019. The knockdown and overexpression efficiencies were verified by qRT-PCR (Additional file 2: Figure S4 D-G). The CCK8 assay showed that FR019089 and FR019019 did not significantly affect cell multiplication, except for a modest increase in HCT8 proliferation upon FR019019 overexpression (Additional file 2: Figure S5). However, clone formation assays did not corroborate this proliferative effect, suggesting FR019019 may not sustain long-term growth advantage. We then interrogated whether they may regulate cell migration and invasion in CRC cells. As illustrated in Fig. 6A,B, the amount of migrated and invasive cells was remarkably cut down after mimic-FR019089 or -FR019019 treatment compared with the mimic-NC group. In contrast, the number of migrated and invasive cells in the inhibitor-FR019089 or -FR019019 group obviously rose (Fig. 6C,D). Collectively, our data show that FR019089 and FR019019 suppress CRC cell motility without altering proliferation, suggesting their role in inhibiting metastatic potential.

Enhanced cell invasion and migration promote cancer metastasis, leading to poor prognosis. We therefore assessed the association of FR019089 and FR019019 with patient survival. Kaplan–Meier analysis revealed that low FR019089 expression was significantly associated with shorter progression-free survival (PFS) and overall survival (OS) in EOCRC (Fig. 6E,F), but not in LOCRC patients (Fig. 6G,H; Additional file 2: Figure S6). Taken together, these results identify FR019089 as a promising prognostic biomarker specific to EOCRC.

### Downstream regulated pathways of FR019089 and FR019019

**Fig. 7:**
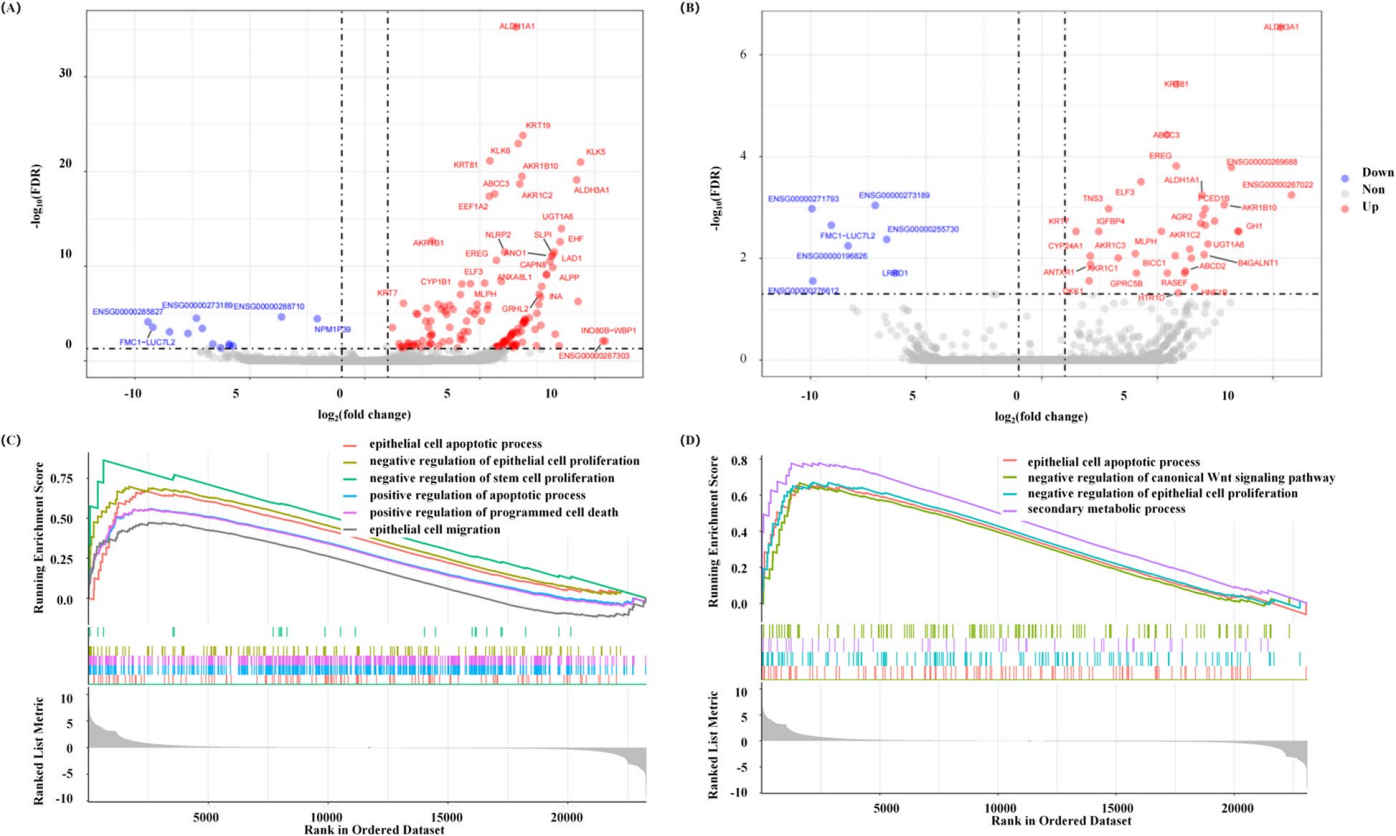
Identification of downstream pathways of FR019089 and FR019019. RKO cells were treated with mimic-FR019089, -FR019019, or -negative control (*n* = 4 for each group). **A,B** Volcano plots illustrate the differentially expressed genes between mimic-negative control with -FR019089 (**A**) or -FR019019 (**B**). Upregulated genes are shown in red and downregulated genes are shown in blue. **C,D** Results of gene-set enrichment analysis for significant pathways related to aggressiveness of colorectal cells in mimic-FR019089 (**C**) and mimic-FR019019 (**D**) group (qvalue < 0.05)

Growing evidence has shown that piRNAs function by forming specific piRNA silencing complexes that bind a diverse spectrum of downstream target genes [41, 42]. We therefore performed RNA-seq to explore putative downstream targets of FR019089 and FR019019. RKO cells were transfected with mimic-FR019089, -FR019019, or negative control. Differential expression analysis revealed 136 upregulated and 13 downregulated genes under FR019089 overexpression, versus 36 upregulated and 7 downregulated genes for FR019019 (Fig. 7A,B). GSEA analysis indicated that mimic-FR019089 transfection promoted the epithelial cell apoptotic process, the positive regulation of programmed cell death, and negative regulation of stem cell proliferation (Fig. 7C and Additional file 1: Table S8), while FR019019 overexpression induced the epithelial cell apoptotic process and the secondary metabolic process, and negative regulation of canonical Wnt signaling pathway (Fig. 7 and Additional file 1: Table S9). Several downstream regulatory molecules (TNF, RIPK3, GATA3, and PPARG) of FR019089 and FR019019 were selected for verification by qRT-PCR. TNF and RIPK3 are involved in triggering programmed cell death, whereas GATA3 and PPARG are strongly associated with suppressing cell proliferation [43–45]. The results of qRT-PCR confirmed that high levels of FR019089 and FR019019 promoted the expression of TNF, RIPK3, GATA3, and PPARG (Additional file 2: Figure S7). These data suggest that FR019089 and FR019019 share partially overlapping functions, with enriched pathways linked to reduced cancer aggressiveness [46–48].

## Discussion

Our findings utilized multi-omics integration to unveil genomic alterations, transcriptomic and epigenomic characteristics, as well as tumor microenvironment and metabolic pathways specific to EOCRC. Notably, integrative analysis of transcriptomic and DNA methylation profiles identified six candidate genes potentially implicated in EOCRC pathogenesis. We observed specific downregulation of PIWIL1, FR019089, and FR019019 in EOCRC, which correlated with increased migration and invasion, as well as poorer survival. Furthermore, in vitro experiments demonstrated the ability of FR019089 and FR019019 to inhibit cell migration and invasion, and subsequent RNA-seq confirmed the downstream anti-oncogenic signaling pathways mediated by high expression of FR019089 and FR019019. These findings provide novel insights into the PIWI/piRNA regulatory axis underlying the aggressive clinical phenotype of EOCRC, and we highlight that FR019089 has potential application in personalized treatment strategies.

In contrast to the declining trend of CRC-related mortality in adults 50 years or older, the overall incidence and mortality of EOCRC have been steadily rising [2, 12]. Emerging evidence supports that EOCRC represents a distinct clinical entity from LOCRC in terms of genetic predisposition, pathological characteristics, and tumor biology. Familial, hereditary, and sporadic EOCRC are three subgroups of EOCRC. Family history and hereditary conditions account for about 30% of EOCRC [49]. Familial adenomatous polyposis and Lynch syndrome are typical ones. For sporadic EOCRC, both environmental factors and genetic susceptibility are responsible for its occurrence, but the related germline genetic variation is unknown. Currently, Wang et al. [41] identified 49 independent genetic loci significantly associated with EOCRC risk, and Alkhateeb et al. [50] found recurring missense mutations in several genes (AXIN2, ALK, CDKN1A, MAP3K1, ROS1, EPCAM, KDM5A, and AURKA) in over 50% of Canadian samples in addition to previously known colorectal cancer-associated genetic variants. Moreover, Laskar et al. [51] sought out two novel risk loci (1p34.1 and 4p15.33) for EOCRC and found novel evidence of probable causal associations for modifiable lifestyle with increased EOCRC risk. EOCRC patients exhibit several common clinic-pathological features, including left colon localization of the primary tumor, more advanced stage at diagnosis, poorer tumor cell differentiation, higher prevalence of signet ring cell histology, and MSI-high due to germline mutations in the DNA mismatch repair [52–55]. Although a lower percentage of patients were considered to have EOCRC in our study (13.40%), we observed more aggressive features of EOCRC, including a higher frequency of stage III and IV at diagnosis and more prevalence of microsatellite instability tumors. These features might be the consequence of a lack of systematic screening in younger patients and more aggressive intrinsic tumor biology [55].

Migration and invasion are two facets of tumor malignancy. Apart from genetic selection, several traits can explain the enhanced aggressiveness of EOCRC. The metabolic shift of tumors to aerobic glycolysis (the Warburg effect) is a well-established hallmark of cancer, and metastatic cells have been shown to increase the uptake, synthesis, and utilization of lipids as a fuel source [56]. Our findings corroborated this paradigm, revealing significant upregulation of both fatty acid oxidation and glycolytic pathways in EOCRC. Moreover, epigenetic regulation, encompassing DNA methylation, histone modifications, and non-coding RNAs, provides a versatile mechanism for dynamic adaptation to microenvironmental inputs [56]. In this study, six EOCRC-specific molecules (PIWIL1, QRFPR, GATA4, CHST8, MS4A1, and WNT3A) exhibited DNA methylation-mediated regulation, consistent with prior reports [57–60]. These molecules likely participate in tumor microenvironment modulation and metabolic reprogramming [61–64]. For instance, PIWIL1 overexpression has been reported to accelerate the growth of HCC tumors through increasing oxygen consumption and energy production via fatty acid metabolism; additionally, PIWIL1-overexpressing HCC cells can attach myeloid-derived suppressor cells (MDSCs) into the tumor microenvironment, which in turn initiate the expression of immunosuppressive cytokine IL10 [39]. However, the precise mechanisms by which PIWIL1 orchestrates metabolic rewiring and immune microenvironment remodeling in CRC remain to be fully elucidated.

PIWIL1 is a member of the PIWI protein family [38]. We observed that PIWIL1 is expressed predominantly in epithelial cells and especially in CMS1 and CMS3. There are marked differences in the intrinsic biological underpinnings between four consensus molecular subtypes: CMS1, hypermutated, microsatellite unstable, strong immune activation; CMS2, epithelial, chromosomally unstable, marked WNT, and MYC signaling activation; CMS3, epithelial, evident metabolic dysregulation; and CMS4, prominent TGF-β activation, stromal invasion, and angiogenesis [65]. Analysis of the CRC tumor microenvironment and cellular metabolism revealed the upregulation of chemokine signaling, antigen processing and presentation, and IL-17 signaling pathways in both EOCRC and LOCRC; meanwhile, a remarkable shift in energy metabolism happened in CRC tissues. Importantly, these changes correlated with elevated PIWIL1 expression in CMS1/CMS3 subtypes, identifying it as a potential therapeutic target.

PIWI proteins and their mediated epigenetic modifications play an essential role in colorectal carcinogenesis and progression [66]. Our study found an increased level of PIWIL1 in CRC tissues relative to normal colonic mucosa. Although PIWIL1 has been reported to drive cancer metastasis in piRNA-independent mechanisms [67], it also functionally cooperates with piRNAs to shape the clinicopathological characteristics of CRC [16]. Emerging evidence suggests the clinical significance of piRNAs in CRC and that genetic variations in piRNAs may modulate CRC susceptibility [14, 68]. However, despite these advances, there is no research on piRNAs specifically related to EOCRC and their association with clinical and pathological phenotypes within this context. Here, novel EOCRC-specific piRNAs (FR019089 and FR019019) were identified, both of which displayed anti-migration and anti-invasion effects, and FR019089 exhibited the potential as a therapeutic molecule. Collectively, our findings contribute to a deeper understanding of the mechanisms of piRNAs in EOCRC tumorigenesis and further underscore the translational value of piRNAs.

However, this study still has several limitations. Firstly, although we endeavored to maximize the inclusion of EOCRC cases to enhance analytical rigor, these results could represent part of the omics characteristic of EOCRC patients due to the older diagnosed age of CRC patients in TCGA and GEO datasets. Secondly, although not reaching statistical significance, we observed potentially important molecular alterations in EOCRC, including lower mutation frequency and copy number variations (deletions/amplifications). Thirdly, the study lacked large-sample and cross-ethnicity external validation of EOCRC in independent datasets, as well as molecular mechanism investigations into PIWIL1/FR019089 and PIWIL1/FR019019, and research on DNA modification. Finally, no significant difference in EOCRC vs. LOCRC for target piRNAs was observed due to the small sample size of CRC patients. Therefore, more in-depth profiling focusing on EOCRC patients and further exploration of mechanisms elaborating EOCRC pathophysiology are of great necessity in future research.

## Conclusions

Our findings characterize the molecular landscape of EOCRC and implicate FR019089 and FR019019 of piRNA as potential modulators influencing the metastatic and invasive characteristics of EOCRC. FR019089 exhibits potential as a predictive biomarker for EOCRC prognosis. To the best of our knowledge, these data provide the first evidence of the role of piRNAs in EOCRC, potentially supporting their use as prognostic biomarkers in EOCRC patients. These findings could be instrumental in advancing precision cancer therapy.

## Supplementary Information

Additional file 1: Table S1. Sequences of qPCR primers. Table S2. Clinical and tumor characteristics of patients with early-onset and late-onset colorectal cancer. Table S3. GSEA analysis of the different expression genes. Table S4. Differentially expressed genes identified in the TCGA database after using inverse probability of treatment weighting method to balance baseline covariates associated with EOCRC and LOCRC. Table S5. Consistent differential methylation sites and transcription genes identified in the TCGA database. Table S6. Baseline information of CRC patients. Table S7. RNA sequences of differentially expressed piRNAs and their IDs in piRBase, piRNAdb and RNAcentral. Table S8. GSEA analysis of RNA-seq results (mimic-FR019089 vs mimic-NC). Table S9. GSEA analysis of RNA-seq results (mimic-FR019019 vs mimic-NC).

Additional file 2: Figure S1. Mutation in APC gene in CRC subgroups. Figure S2. The Spearman correlation of DNA methylation with gene expression. Figure S3. Expression pattern of PIWIL1 in different cell types and CMSs of age-of-onset colorectal cancer. Figure S4. Basal expression of FR019089, FR019019, and FR132045 in colon cell lines and efficiency verification of mimic and inhibitor. Figure S5. Effects of FR019089 and FR019019 on cellular proliferation. Figure S6. The prognostic significance of FR019019 in CRC patients. Figure S7. FR019089 and FR019019 promote the expression of TNF, RIPK3, GATA3, and PPARG.

## Data Availability

The data that support the findings of this study are publicly available as described in the Method section or available from the corresponding author upon reasonable request. Analysis codes used to generate the results were deposited to the GitHub repository (https://github.com/yunyun-1996/EOCRC.git).

## Abbreviations

CRC: Colorectal cancer
EOCRC: Early-onset colorectal cancer
LOCRC: Late-onset colorectal cancer
ncRNA: Non-coding RNA
piRNA: PIWI-interacting RNA
PIWIL1: Piwi Like RNA-Mediated Gene Silencing 1
MSI: Microsatellite instability
ssGSEA: Single sample gene set enrichment analysis
DEGs: Differentially expressed genes
DMCs: Differentially methylated CpGs
QRFPR: Pyroglutamylated RFamide peptide receptor
CHST8: Carbohydrate sulfotransferase 8
GATA4: GATA binding protein 4
MS4A1: Membrane spanning 4-domains A1
WNT3A: Wnt family member 3A
NES: Normalized enrichment score
PFS: Progression-free survival
OS: Overall survival
GEO: Gene Expression Omnibus
FC: Fold change
FDR: False discovery rate
KEGG: Kyoto Encyclopedia of Genes and Genomes
t-SNE: *t*-Distributed stochastic neighbor embedding
UMI: Unique molecular identifier
SD: Standard deviation
GO: Gene ontology
IPTW: Inverse probability of treatment weighting
