# Supplementary material for: Integration of multi-omics data to unveil the molecular landscape and role of piRNAs in early-onset colorectal cancer
Source: BMC Med. 2025 Apr 29;23:250. doi: 10.1186/s12916-025-04074-2 (PMC12042543; doi:10.1186/s12916-025-04074-2)
Supplement: Supplementary file 2 — Additional file 2: Figure S1. Mutation in APC gene in CRC subgroups. Figure S2. The Spearman correlation of DNA methylation with gene expression. Figure S3. Expression pattern of PIWIL1 in different cell types and CMSs of age-of-onset colorectal cancer. Figure S4. Basal expression of FR019089, FR019019, and FR132045 in colon cell lines and efficiency verification of mimic and inhibitor. Figure S5. Effects of FR019089 and FR019019 on cellular proliferation. Figure S6. The prognostic significance of FR019019 in CRC patients. Figure S7. FR019089 and FR019019 promote the expression of TNF, RIPK3, GATA3, and PPARG. [file 12916_2025_4074_MOESM2_ESM.docx]

**Supplementary Figures**

**
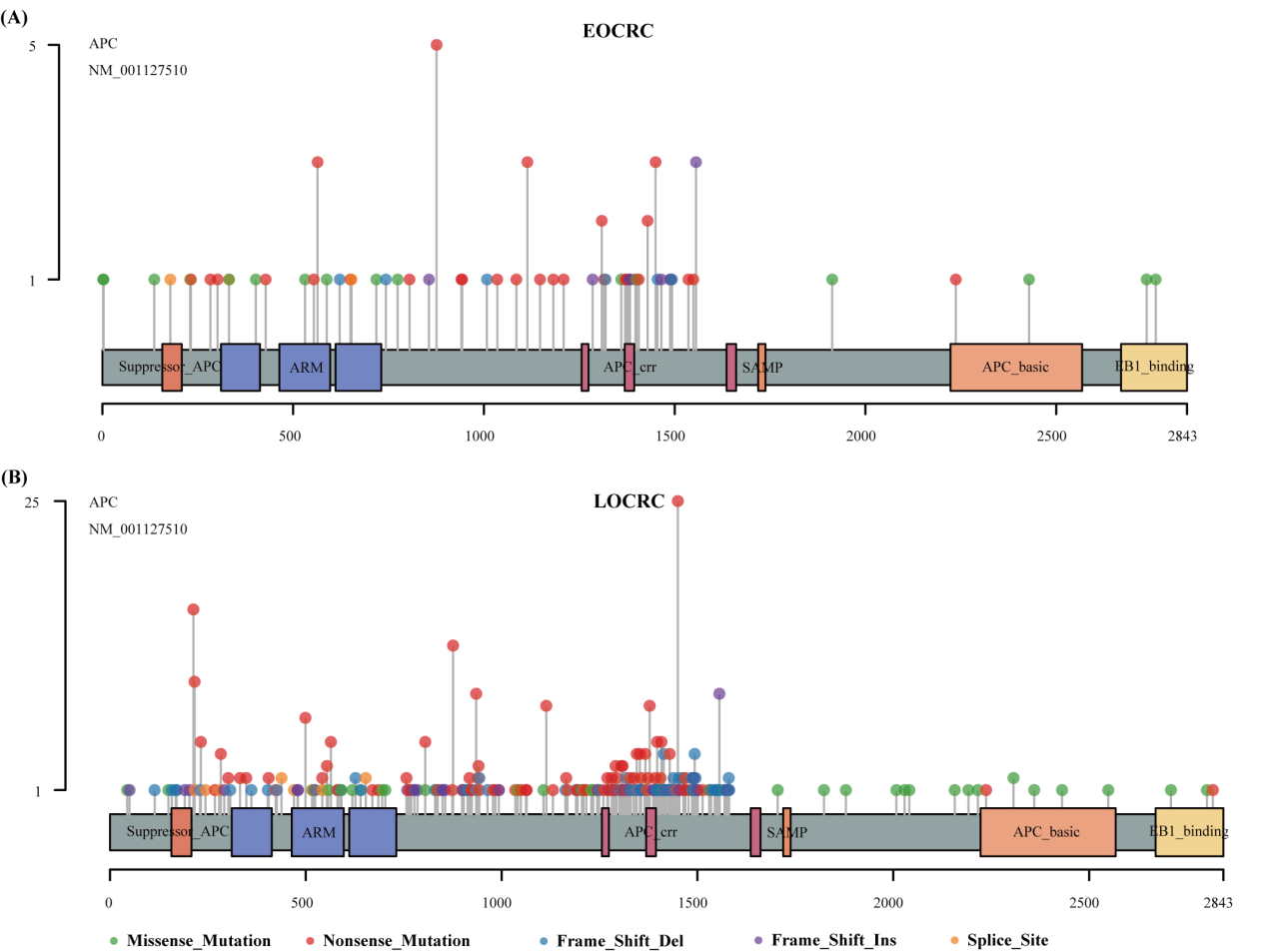
**

**Figure S1. Mutation in *APC* gene in CRC subgroups.** The lollipop charts show the distribution of mutation spots and mutation types of the *APC* gene in early-onset colorectal cancer (**A**) and late-onset CRC (**B**).

**
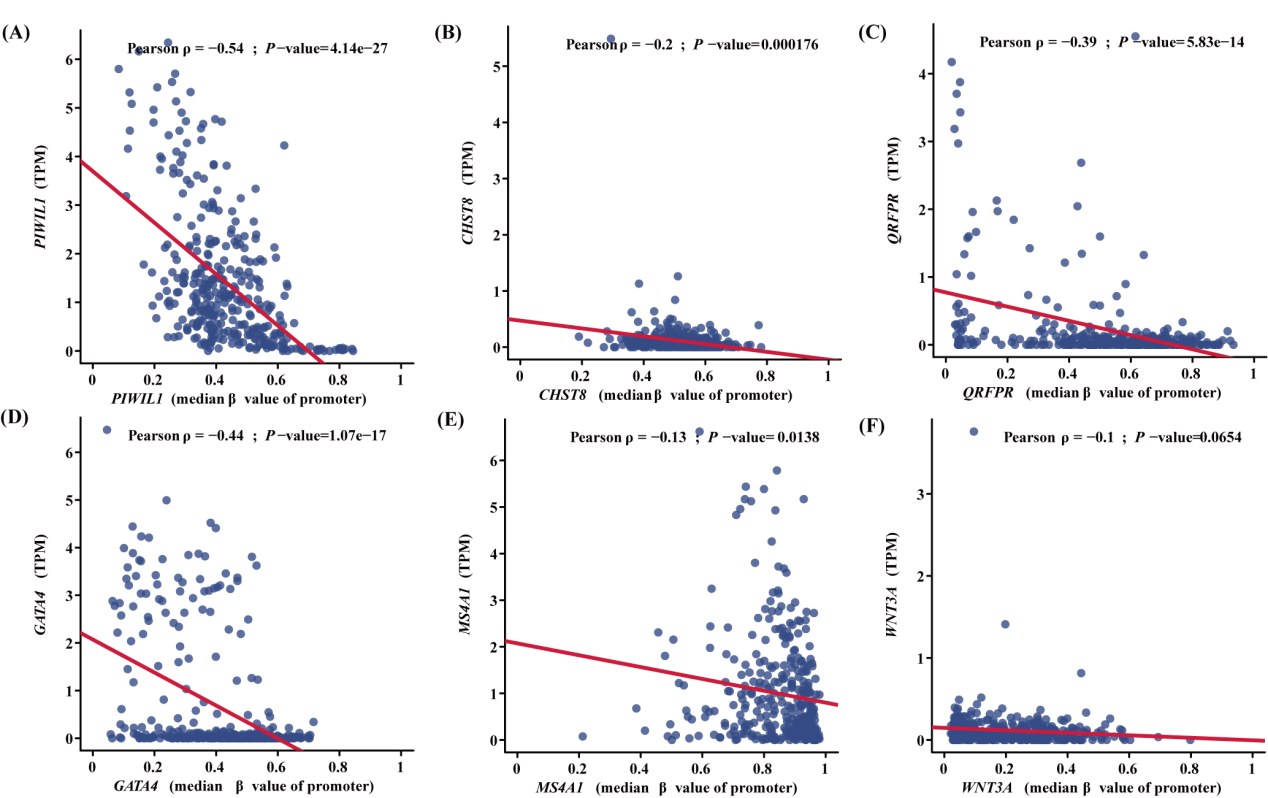
**

**Figure S2.** **The Spearman correlation of DNA methylation with gene expression.** Matched methylation and gene expression data from The Cancer Genome Atlas were plotted to illustrate the correlation for selected methylation-related genes: *PIWIL1* **(A)**, *CHST8* **(B)**, *QRFPR* **(C)**, *GATA4* **(D)**, *MS4A1* **(E)**, and *WNT3A* **(F)**. The x-axes give the β value of the region of the MRG 200 bp upstream of the transcription start site as a degree of methylation. The y-axes give the gene expression as log_2_transformed 1+reads per kilobase of transcript per million mapped reads values of RNAseq.


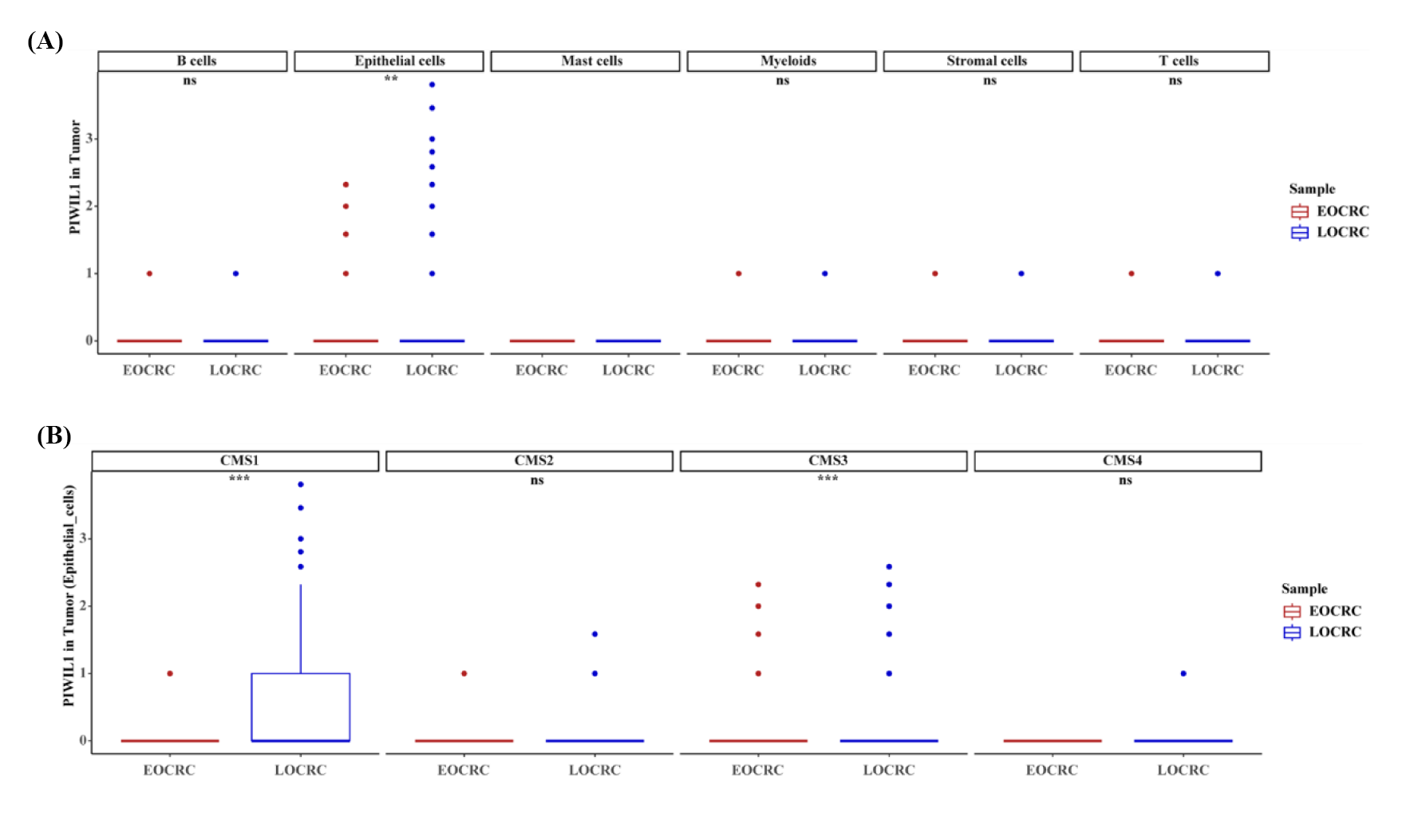


**Figure S3.** **Expression pattern of *PIWIL1* in different cell types and CMSs of age-of-onset colorectal cancer. A**, Scatterplots indicate differential expression of *PIWIL1* in six cell types of EOCRC and LOCRC tumors. **B**, Differential expression of *PIWIL1* in tumor epithelial cells across four CMSs. *P* values were calculated *via* the Wilcoxon rank-sum test. ***P*<0.01 and ****P*<0.001. *ns*, no significance; EOCRC, early-onset colorectal cancer; LOCRC, late-onset CRC; CMS, consensus molecular subtype.

**
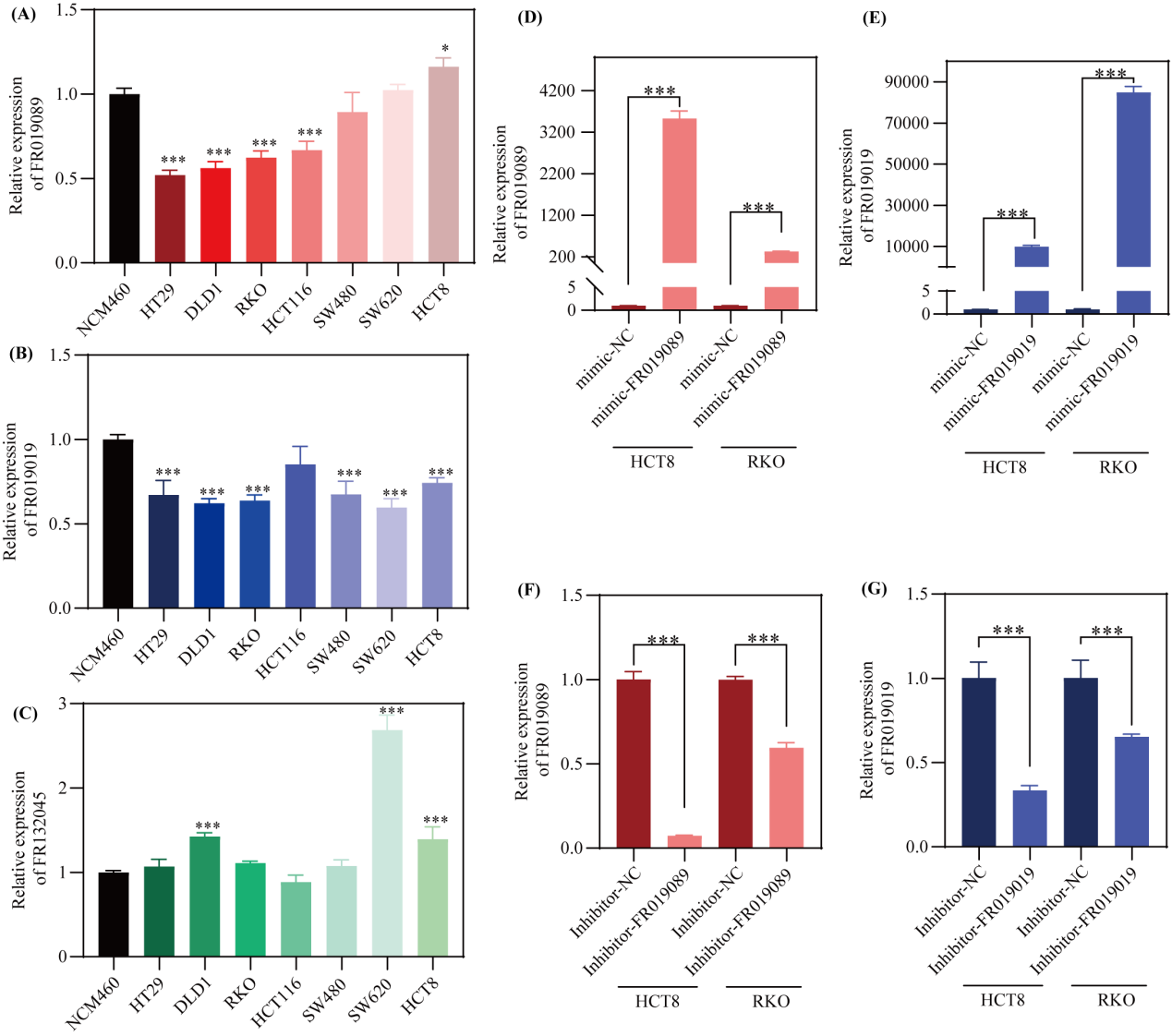
**

**Figure S4. Basal expression of** **FR019089, FR019019, and FR132045 in colon cell lines and efficiency verification of mimic and inhibitor.** **A-C**, qRT-PCR detection of FR019089 **(A)**, FR019019 **(B)**, and FR132045 **(C)** in NCM460, HT29, DLD1, RKO, HCT116, SW480, SW620, and HCT8 cells. U6 was used as an internal control. The results are presented as the mean±SD. Statistical analyses were performed by one-way ANOVA with Dunnett’s test. **P*<0.05, ****P*<0.001 vs. the NCM460 group. **D-E**, qRT-PCR detection to test the over-expression efficiency of mimic-FR019089 **(D)** and -FR019019 **(E)** in HCT8 and RKO cells. **F-G**, qRT-PCR detection of FR019089 and FR019019 in HCT8 and RKO cells after inhibitor-FR019089 **(F)** and -FR019019 **(G)** treatment. U6 was used as an internal control. The results are presented as the mean±SD. Statistical analyses were performed by two-tailed Student’s t-test. ****P*<0.001 *vs.* the mimic-NC or inhibitor-NC group.

**
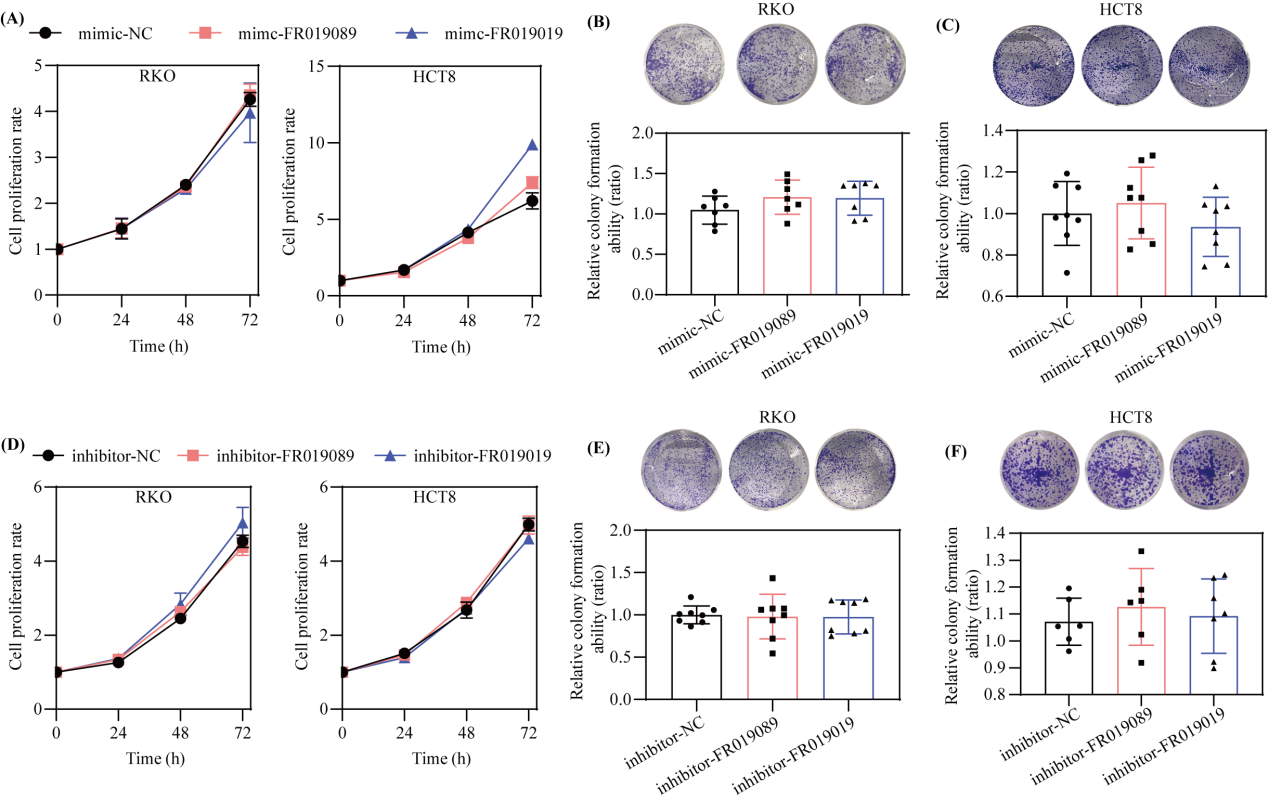
**

**Figure S5. Effects of FR019089 and FR019019 on cellular proliferation. A-B**, RKO and HCT8 cells were transfected with either mimic-NC, -FR019089, or -FR019019. The treated cells were subsequently used for CCK8 assay **(A)** and colony formation assay **(B, C)**. **D-F**, RKO and HCT8 cells were transfected with either inhibitor-NC, -FR019089, or -FR019019. The treated cells were subsequently used for CCK8 assay **(D)** and colony formation assay **(E, F)**. The results are presented as the mean±SD. Statistical analyses were performed by one-way ANOVA with Dunnett’s test.

**
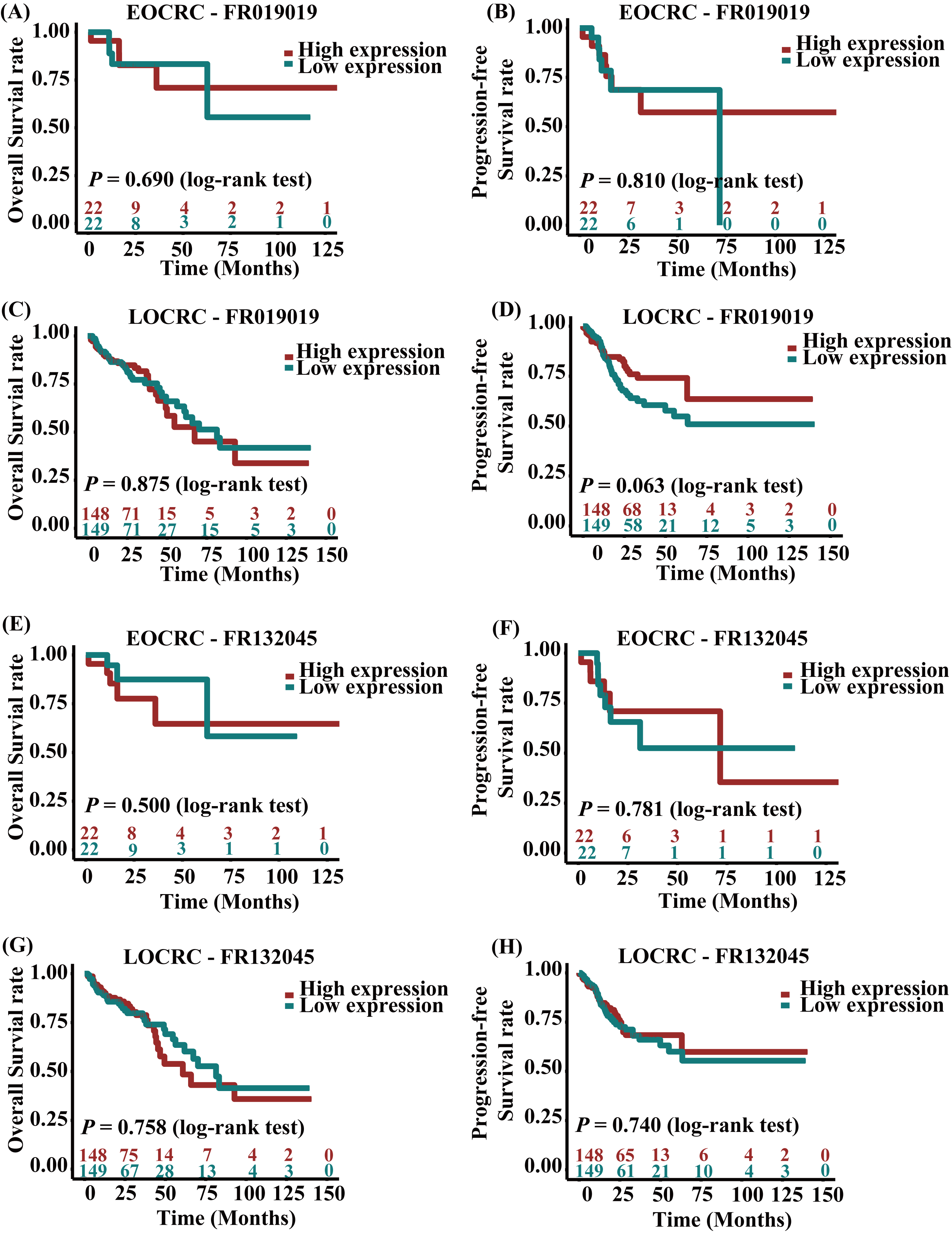
**

**Figure S6. The prognostic significance of FR019019 in CRC patients. A-D,** The overall survival and progression-free survival analysis was performed by Kaplan-Meier test and log-rank method in EOCRC **(A, B)** and LOCRC **(C, D)** patients respectively. EOCRC, early-onset colorectal cancer; LOCRC, late-onset CRC.


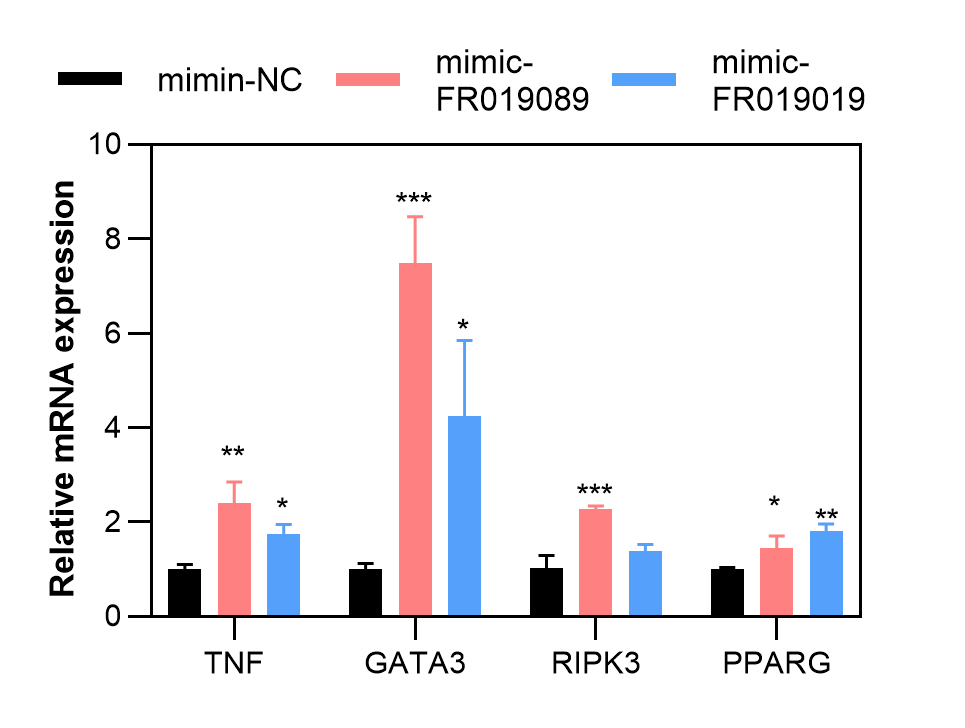


**Figure S7. FR019089 and FR019019 promote the expression of TNF, RIPK3, GATA3, and PPARG.** qRT-PCR detection of TNF, RIPK3, GATA3, and PPARG mRNA in HCT8 cells. U6 was used as an internal control. The results are presented as the mean±SD. Statistical analyses were performed by one-way ANOVA with Dunnett’s test. *P<0.05, ***P<0.001, ***P<0.001 vs. the mimic-NC group.
